# Dual-Enzyme-Based Signal-Amplified Aptasensor for Zearalenone Detection by Using CRISPR-Cas12a and Nt.AlwI

**DOI:** 10.3390/foods11030487

**Published:** 2022-02-08

**Authors:** Xijing Yao, Qingli Yang, Yifei Wang, Chuanlin Bi, Han Du, Wei Wu

**Affiliations:** 1 College of Food Science and Engineering, Qingdao Agricultural University, No. 700 Changcheng Road, Qingdao 266109, China; 20192105241@stu.qau.edu.cn (X.Y.); yql@qau.edu.cn (Q.Y.); qetuoadgjlp@126.com (Y.W.); duhan1211@163.com (H.D.); 2 Qingdao Institute of Special Food, No. 700 Changcheng Road, Qingdao 266109, China; 3 Qingdao Institute for Food and Drug Control, No. 7 Longde Road, Qingdao 266010, China; 007664@163.com; 4 College of Food Science and Engineering, Shandong Agricultural University, Taian 271018, China

**Keywords:** CRISPR biosensor, Nt.AlwI nicking endonuclease, ZEN, signal amplification, nucleic-acid detection

## Abstract

Zearalenone (ZEN) is harmful to animals and human beings, so it is very important to develop a rapid and sensitive method for the detection of ZEN. In this paper, we proposed a novel ZEN-monitoring method using two aptamers as recognition elements and EnGen LbaCas12a and Nt.AlwI nicking endonuclease as signal amplifiers. When ZEN was present, it bound to the aptamer Z0 and, Z1 was released into solution. The solution was then separated and the Nt.AlwI enzyme was added in order to form a nicking-enzyme cycle, thereby producing large amounts of the ssDNA Z3 for 30 min. The Z3 formed a CRISPR-Cas12a-Z3 complex with CRISPR-Cas12a, activated the trans-cleavage ability of Cas12a, cleaved the Quenched Reporter for 20 min, and underwent fluorescence recovery. The aptasensor was able to sensitively detect ZEN in the linear range of 1–1000 pg/mL, with a detection limit as low as 0.213 pg/mL. The detection time lasted for 2 h. Additionally, this detection technology can also be used to monitor other hazards.

## 1. Introduction

Zearalenone (ZEN) is a nonsteroidal estrogenic mycotoxin produced primarily by *Fusarium graminearum* and is commonly found in wheat, corn, sorghum, barley, soybeans and other grains as well as dairy products [1]. ZEN in contaminated agricultural and livestock products can accumulate in the human body through the food chain, which poses a great threat to human health. Studies have shown that ZEN has strong mutagenicity, teratogenicity, neurotoxicity, reproductive toxicity and carcinogenic effects on animals and humans [2]. Therefore, it is necessary to take necessary preventive measures. ZEN exposure of more than 10 ug/kg can cause serious disease [3]. At present, there is no specific medicine to treat ZEN poisoning in animals; ZEN residues accumulate in the body and the time for the general toxin to be metabolized out of the body is as long as half a year. Therefore, effective, sensitive, and specific methods are needed to detect and monitor ZEN. Although many instrumental and bioanalytical methods have been established to detect mycotoxin contamination, their high cost, complex operation level and laboratory and environmental requirements limit their widespread application [4]. At present, the main methods of ZEN detection in China involve instrumental detection and immunoassay. Generally speaking, the instrument-detection method [5] has a high detection accuracy and good selectivity, but it often needs a complex pretreatment process, high-cost detection instruments and equipment, standardized operations of professionals, long detection time, and is not suitable for the needs of immediate and rapid detection. Immunoassay is simple and fast. However, it has the disadvantages of poor stability, poor affinity for small molecular targets, and high price relative to the price of aptamers [6].

An aptamer is an oligonucleotide sequence (DNA or RNA). It is usually obtained from a library of nucleic-acid molecules by an in vitro screening technique, i.e., the systematic evolution of ligands by exponential enrichment (SELEX) [7]. Nucleic-acid aptamers are widely used in the field of biosensors because they can bind to a variety of target substances with high specificity and selectivity [8,9,10,11,12]. Our group has also reported several aptamer-based relative-detection methods [13]. The aptamer has the advantages of a short screening period, low cost, easy synthesis, stability and long-term preservation, and a high affinity and specificity.

DNA-cleaving endonucleases are a special class of restriction endonucleases that recognize mismatched sequences in hybridized double-stranded DNA and hydrolyze only one target strand [14]. Yang et al. reported a DNA nanomachine constructed of DNA-functionalized gold nanoparticles (DNA-AuNP) powered by a nicking endonuclease, which can move a DNA walker along a three-dimensional (3D) DNA-AuNP trajectory and perform the task of releasing a payload [1]. Xu et al. designed an ultrasensitive and selective colorimetric DNA detection using nicking-endonuclease-assisted nanoparticle amplification. Zou et al. designed an invasive response combined with nicking-endonuclease-assisted nanoparticle amplification for sensitive and specific colorimetric DNA detection [15]. Signal-amplification technology is used to amplify the target-specific signal, rather than the target itself, which does not cause cross-contamination of amplification, so it is more suitable for the detection of harmful substances.

The CRISPR/Cas system, consisting of clusters of regularly spaced, short palindromic repeats (CRISPRs) and CRISPR-associated proteins (Cas), is an adaptive immune system found in bacteria and archaea that naturally protects against invading nucleic acids [16]. CRISPR/Cas systems have made great strides in the field of biotechnology, and they have become an important tool for transcriptional regulation and genome editing [17,18,19,20]. CRISPR/Cas12a (Cpf1), a class 2 V-a CRISPR-related enzyme, not only cleaves the target DNA but also exhibits additional cleavage activity (also referred to as trans-cleavage activity) after cleavage of the target DNA whereby it non-specifically cleaves any non-target ssDNA [21,22]. This collateral cleavage activity can degrade nucleic-acid-reporter molecules thousands of times per second, thereby significantly improving the detection sensitivity of many targets. So far, there have been many detection methods using the CRISPR/Cas enzyme system to verify expression [23,24]. For example, in the field of medical nucleic-acid diagnosis, CRISPR/Cas13a has been used for the rapid and specific quantification of various low-expression RNAs in early cancer [25], as an electrochemiluminescence biosensor for the target-free amplification of human-papillomavirus-subtype (HPV-16) DNA [26], for the detection of ctDNA based on an entropy-driven, strand-displacement reaction (ESDR) triggered by CRISPR/Cas9 cleavage, and as an ultrasensitive electrochemical biosensor for listeria monocytogenes based on CRISPR/Cas12a [27].

In this work, we first introduced a fluorescence aptasensor based on the dual signal amplification of EnGen LbaCas12a and Nt.AlwI nicking endonuclease. Recently, there have been many biosensor applications of the CRISPR/Cas system, but most of them directly utilized the cis-cleavage and trans-cleavage activities of the CRISPR/Cas system. Its applications are often limited to PAM sequences, which are of a single type and have a low detection limit. Therefore, we designed a fluorescence-detection-and-analysis method using a double-enzyme module to greatly improve the detection limit. First, the aptamer’s excellent selectivity was utilized to capture specific targets. Then, a large number of EnGen LbaCas12a PAM sequences (Z3) were amplified by using the Nt.AlwI nicking endonuclease‘s cutting properties. Finally, the fluorescence-detection method of fluorescence-signal recovery was implemented by using the Cas12a-crRNA-Z3 system’s efficient trans-cutting characteristics and a rapid-shear Quenched Reporter.

## 2. Materials and Methods

### 2.1. Reagents and Materials

Monodispersed Magnetic Streptavidin Microspheres (MBs) were purchased from Tianjin Bessel Chromatography Technology Development Center (Tianjin, China), with a solid content of 0.5% (*w/v*) and a particle size of 300 nm. ZEN, Ochratoxin a (OTA), Fumonisin (FB1), Aflatoxin B1 (AFB1) and Aflatoxin M1 (AFM1) were bought form Meizheng Testing Technology Co., Ltd. (Beijing, China). EnGen LbaCas12a from *Lachnospiraceae bacterium ND2006* was purchased from New England Biolabs (Ipswich, MA, USA). CRISPR RNA (crRNA) and other ssDNAs were purchased from Sangon Biotech Co., Ltd. (Shanghai, China). Sequence details are given in Table S1. The buffer solution 1 was used as a reaction solution for the streptavidin-modified magnetic beads and DNA. The CutSmart Buffer and NEBuffer™ 2.1 was from New England Biolabs. Buffer components are visible in Table S2. PBS, agarose, 50 × TAE, 5 × TBE, EDTA, 30% acrylamide solution (ACR-BIS, 29:1), tetramethylethylenediamine (TEMED), PAGE coagulant, ammonium persulfate (APS)^+^, 6× loading buffer and DNA Marker were purchased from Sangon Biotechnology Co., Ltd. (Shanghai, China). RNase-free water was employed as the solvent throughout the experiment. All chemical reagents were of analytical grade.

### 2.2. Apparatus

Fluorescence detection was performed using the F-2700 fluorescence spectrophotometer (Hitachi Ltd., Tokyo, Japan). Gel electrophoresis experiments were performed using a Mini-Sub Cell GT System (Bio-Rad Laboratories, Lnc., Hercules, CA, USA). The secondary structure changes of nucleic acids were detected by circular-dichroism (CD) spectroscopy (Applied Photophysics Ltd., Leatherhead, Surrey, UK). The Zeta-Potential image was measured by a Malvern Dynamic Scatterometer (Malvern Instruments Ltd., Enigma Business Park Grovewood Road, Malvern, Worcestershire WR141XZ, UK). Double-stranded DNA was synthesized by a PCR instrument (Biometra, Göttingen, Germany). The amount of binding of the magnetic beads to the nucleic acid was measured by an Evolution 201 UV-Vis spectrophotometer (Thermo Fisher Scientific Co Ltd., Beijing, China)

### 2.3. Assembly of Double-Stranded DNA Z0Z1

DNA Z0Z1 was assembled from two single-stranded DNA (Z0 and Z1), wherein the ssDNA Z0 was an aptamer (the K_d_ value is 15.2 ± 3.4 nM) [28] for ZEN and the 5′ end was modified by biotin, and the Z1 contained a Nt.AlwI-nicking-endonuclease recognition site. Z0 contained 62 bases and Z1 contained 18 bases. Among them, Z1 and Z0 contained 12 consecutive complementary pairs.

The buffer was 0.1 M NaCl, 10 mM Tris-HCl. The PCR synthesis program was set to unwind the DNA by heating at 95 °C for 10 min, then the temperature was set to 37 °C and the mixture was incubated for 30 min. The assembly results of Z0Z1 can be verified by 3% agarose electrophoresis (110 V, 25 min).

### 2.4. Fabrication of MB-Z0Z1 Recognition Probe

A solution of 100 μL of MB was separated from the solution using magnetic racks and then washed twice with PBS buffer. The prepared DNA Z0Z1 was mixed and dispersed in buffer 1 (10 μM). MBs were thoroughly mixed with the biotin-modified aptamer Z0Z1 for 30 min. Z0Z1 was attached to the MBs. After that reaction was completed, the MB-Z0Z1 identification probe was obtained and subjected to magnetic separation. The MB-Z0Z1 was washed with PBS buffer 3 times, and finally the MBs were stored in PBS buffer at 4 °C for later use.

### 2.5. Specific Recognition of ZEN Toxin

The prepared ZEN-toxin-capture probes MB-Z0Z1 were added to a centrifuge tube containing a sample of solution to be tested, at a total volume of 100 μL. Because the Z1 oligonucleotide only has a partial base, the Z0 and Z1 aptamers complement each other, and the affinity of the Z0 aptamer for the ZEN toxin is better than the affinity of the Z0 aptamer for the Z1 oligonucleotide, so the Z1 oligonucleotide can dissociate from the Z0 aptamer into solution. The centrifuge tube was placed on a rotary mixer and reacted for 30 min. After the sufficient reaction of ZEN with MB-Z0Z1, ZEN combined with Z0 to form MB-Z0-ZEN and released Z1. The solution was magnetically separated to obtain a supernatant containing Z1, which was kept at 4 °C for later use.

### 2.6. Fabrication of MB-Z2 Amplification Probe

A solution of 100 μL of MBs was separated using magnetic racks and then washed twice with PBS buffer. The Z2 ssDNA was mixed and dispersed in buffer 1 (10 μM). MBs were mixed with that solution and vortexed at room temperature for 30 min to allow biotin to bind well to streptavidin. After that reaction was finished, the MB-Z2 amplification probe was obtained, magnetic separation was carried out, magnetic beads were washed by PBS buffer solution three times, and finally the magnetic beads were stored in the PBS buffer solution and stored at 4 °C for later use.

### 2.7. Nt.AlwI Enzyme Digestion Preliminary Signal Amplification

The supernatant containing Z1 was added to 100 μL (excess) of prepared MB-Z2, vortexed and mixed, and reacted for 10 min in a gas-bath shaker at room temperature. Z1 hybridized with Z2 of the Z2-MB through base complementation to form a double chain, and the MB-Z2Z1 complex was obtained.

Nt.AlwI is a nicking endonuclease that cleaves only one strand of DNA on a double-stranded DNA substrate. It is an engineered derivative of AlwI, which catalyzes a single strand break 4 bases beyond the 3′ end of the recognition sequence on the top strand. When the enzyme completes the shearing, the short Z1 oligonucleotide strand is too unstable to bind to the severed Z2 strand, and the free Z1 strand that dissociates from the Z2 strand continues to be sheared by the Nt.AlwI enzyme after combining with other Z2 strands.

An amount of 1 μL of Nt.AlwI enzyme (1 μM) was added to the MB-Z1Z2 solution, the buffer was CutSmart Buffer, and the solution volume was 100 μL. After the solution was mixed, the reaction was carried out at room temperature and was shaken for a few minutes. This solution was magnetically separated to obtain a solution containing Z3. This solution was rapidly cooled to 4 °C after inactivating the enzyme at 80 °C for 20 min.

### 2.8. The Cas12a-crRNA Duplex

The Cas12a and crRNA were mixed in a ratio of 1:1 in 1 × NEBuffer™ 2.1 and reacted for 10 min at room temperature to form the Cas12-crRNA complex.

### 2.9. Secondary Signal Amplification of CRISPR-Cas12a

The prepared Cas12a-crRNA complex was added to the supernatant containing Z3 and mixed. The reaction was allowed to stand for 10 min at room temperature to form a complex of Cas12a-crRNA-Z3, thereby activating the trans-cleavage activity of the enzyme. The Quenched Reporter (a fluorescent self-quenching molecule consisting of 10 A bases of FAM and a BHQ that was modified at both ends) was added to the reaction system as a substrate for the enzymatic trans-cleavage of Cas12a-crRNA-Z3. The buffer was NEBuffer 2.1. The solution was diluted to 300 μL with enzyme-free water. The solution was reacted at 37 °C for 20 min to complete the second signal amplification. Subsequently, the fluorescence intensity was scanned with an F-2700 fluorescence spectrophotometer, and the fluorescence intensity at the emission wavelength of 517 nm was measured at the excitation wavelength of 495 nm.

## 3. Results and Discussion

### 3.1. Principle of the Cas12a-Based Biosensor

**Figure 1:**
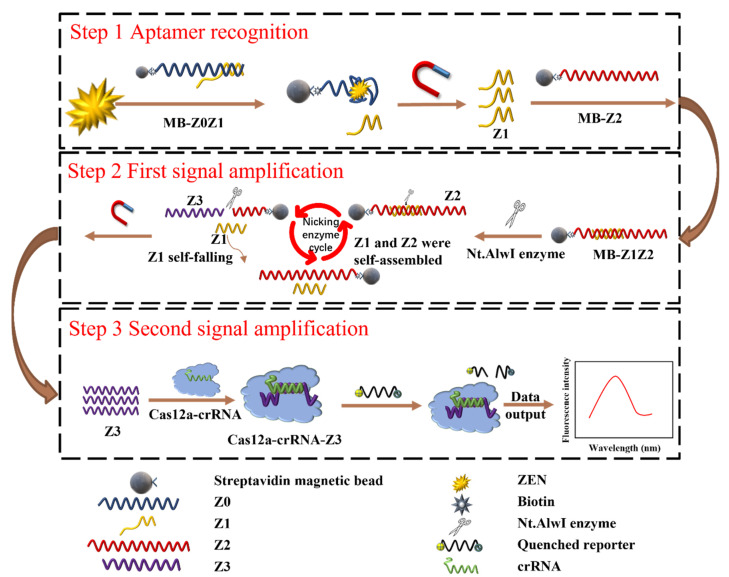
Schematic diagram of a magnetic-bead-assisted dual-signal-amplification aptasensor for sensitive ZEN detection based on the Nt.AlwI enzyme and the Cas12a enzyme. Step 1: The aptamer probe recognizes the ZEN toxin and causes Z1 to dissociate into solution by competitive binding. Step 2: After Z1 and Z2 were hybridized, the cutting activity of the Nt.AlwI enzyme was activated, the Z2 chain was cut to release Z3, Z1 was self-shed after the cutting was finished and it hybridized with Z2 again, and a large amount of Z3 was released by the enzyme-cutting signal amplification to achieve the first signal amplification. Step 3: The combination of Z3 and the Cas12a-crRNA complex activates trans-cleavage activity, non-specifically cleaving any ssDNA so that the added fluorescent signal molecule was cleaved and the quenched fluorescence was restored.

In this work, a fluorescent signal-amplified aptasensor based on magnetic beads, aptamers and enzyme digestion was proposed for ZEN detection. The detection principle of the ZEN aptasensor is shown in Figure 1. First, two partially complementary ssDNAs (Z0, Z1) self-assemble to form a detection probe according to the principle of base complementarity. Secondly, the detection probe and magnetic beads were assembled into MB-Z0Z1. In the same operation, Z2 and MB were made into the amplification probe MB-Z2.

The scheme is shown in Figure 1. When ZEN was present, ZEN would bind competitively with the aptamer Z0, allowing Z1 to be released into solution. The solution was subjected to magnetic separation to separate the excess MB-ZOZ1 and MB-Z0-Zen complexes. A supernatant containing Z1 was thus obtained (Step 1).

The amplification probe MB-Z2 was added into the supernatant, two ssDNAs (Z1 & Z2) formed a dsDNA, while the Nt.AlwI enzyme recognized the dsDNA and cleaved only the Z2 ssDNA. After enzyme digestion, the Z3 ssDNA and Z1 ssDNA detached from the magnetic beads, and the detached Z1 ssDNA continued to combine with the other Z2 ssDNA to continue the reaction. The solution was subjected to magnetic separation to separate the excess MB-Z2 and MB-Z2-Z1 complexes. A supernatant containing Z3 was thus obtained. This was the first signal amplification (Step 2).

The prepared Cas12a-crRNA complexes were added to the solution containing Z3 ssDNA, and they reacted at room temperature for 10 min to form Cas12a-crRNA-Z3 complexes. The study by Li et al. showed that only the RuvC domain catalyzes the pocket exposure to the two-lobe structure of the terpolymer, thus allowing the trans-cleavage of ssDNA. The quenched fluorescent ssDNA was added to the solution to be non-specifically cleaved by the Cas12a-crRNA-Z3 complex. Through the effect of fluorescence recovery, the second signal amplification was achieved (Step 3).

### 3.2. Characterization of dsDNA

The solution consisted of 10 μL of 20 μM Z1 ssDNA and 10 μL of 20 μM Z0 ssDNA. Then, 2 μL of 10× hybridization salt buffer (1 M NaCl, 100 mM Tris-HCl) was added to the solution and 8μL of ultrapure water was added in order to dilute the solution. Finally, the solution was subjected to nucleic-acid hybridization by PCR. The PCR synthesis program was set to unwind the DNA by heating at 95 °C for 10 min, then the temperature was set to 37 °C and the mixture was incubated for 30 min. The assembly results of Z0Z1 can be verified by 3% agarose electrophoresis (110 V, 25 min). The Z1 ssDNA and Z2 ssDNA were characterized by the same method.

**Figure 2:**
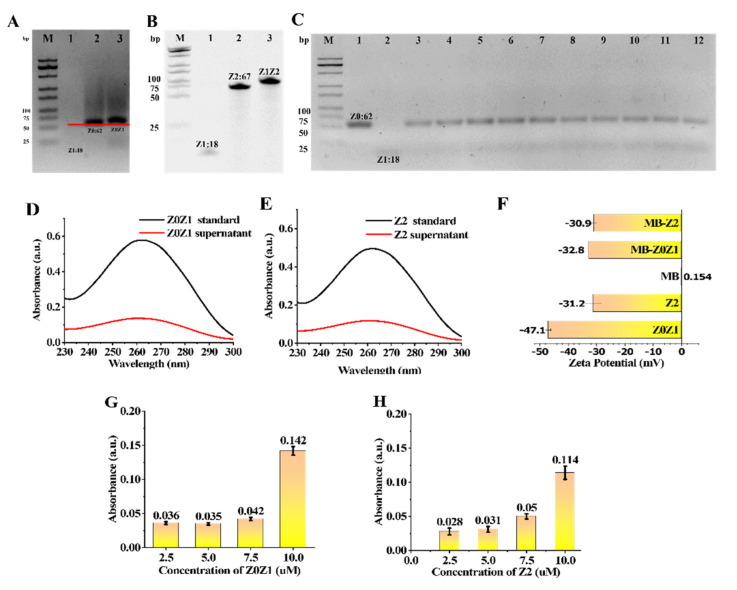
Various characterization optimization of nucleic-acid strands Z0Z1 and Z2. (**A**) DNA self-assembly was characterized by 3% agarose gel electrophoresis (80 V, 25 min). Lane M: DNA marker, lane 1: ssDNA Z0 and ssDNA Z1, lane 2: dsDNA Z0Z1. (**B**) DNA self-assembly was characterized by 10% polyacrylamide electrophoresis (110 V, 80 min). Lane M: DNA marker, lane 1: Z1, lane 2: Z2, lane 3: Z1Z2. (**C**) DNA self-assembly was characterized by 3% agarose gel electrophoresis (80 V, 25 min). Lane M: DNA marker, lane 1: Z0, lane 2: Z1, lane 3: Z0:Z1 = 1:0.8, lane 4: Z0:Z1 = 1:0.85, lane 5: Z0:Z1 = 1:0.9, Lane 6: Z2:Z2 = 1:0.95, lane 7: Z0:Z1 = 1:1; Lane 8: Z0:Z1 = 1:1.05, Lane 9: Z0:Z1 = 1:1.1, Lane 10: Z0; Z1 = 1:1.15, Lanes 11: Z0:Z1 = 1:1.2, and Lane 12: Z0:Z1 = 1:1.25. (**D**) UV-Vis absorption spectra of supernatant before and after binding of magnetic beads with DNA Z0Z1. (**E**) UV-Vis absorption spectra of supernatant before and after binding of magnetic beads with DNA Z2. (**F**) Zeta Potentials of the MB, DNA Z0Z1, DNA Z2, MB-Z0Z1 and MB-Z2. (**G**) UV-Vis absorption spectra of Z0Z1 standard (black curve) and the supernatant after Z0Z1 reacted with MB (red curve). (**H**) UV-Vis absorption spectra of Z2 standard (black curve) and the supernatant after Z2 reacted with MB (red curve).

To verify whether the designed ssDNA can be successfully combined into the required dsDNA, electrophoresis was carried out. The position of Z0Z1 in lane two was slightly higher than that of Z0 in lane one (Figure 2A). Therefore, the successful binding of Z1 and Z0 can be verified. The position of Z1Z2 in lane three was slightly higher than that of Z2 in lane two (Figure 2B). Therefore, the successful binding of Z1 and Z2 can be verified.

To obtain the best combination ratio of Z0 and Z1, ten groups of Z0 + Z1 mixed reactions with different concentration ratios were established. The concentration of Z0 in each lane was 5 μM and different concentrations of Z1 were added for the hybridization reaction. Ultrapure water was used to adjust the concentration of the solution. The final concentration of the buffer was 0.1 M NaCl and 10 mM Tris-HCl. With the increase in the proportion of Z1, the yield of assembled DNA increased gradually. Z0 and Z1 had the highest synthesis efficiency when the concentration ratio was 1:1.1, so it was selected as the best optimized concentration (Figure 2C).

### 3.3. Characterization of Nucleic Acids Coupled to MB

The formation of the bead-nucleic-acid structure was verified by UV absorption and absorbance spectra of the Zeta Potential. In the UV-Vis spectrum (Figure 2G,H) DNA has a prominent characteristic absorption peak at 260 nm. First, a nucleic-acid standard diluted with 100μL buffer 1 was prepared as the Z0Z1 standard and the Z2 standard, respectively, and the characteristic absorption peaks of the nucleic acid were measured using a micro cuvette and the reading was recorded (Figure 2D,E black line). Then, an equal amount of MB was added to the two standard solutions and reacted for 30 min at room temperature, then separated by magnetic separation. The characteristic absorption peaks of nucleic acid in the two supernatants were measured and the readings were recorded. Z0Z1 and Z2 had a significant drop at 260 nm (Figure 2D,E red line). As shown in Figure 2F, the absorbance spectra of the MB, Z0Z1 DNA, Z2 DNA, MB-Z0Z1 and MB-Z2 were 0.15 mV, −47.07 mV, −31.20 mV, −32.77 mV and −30.90 mV, respectively. This phenomenon describes the coupling of negatively charged DNA to the surface of MBs. In general, both methods demonstrate the successful assembly of magnetic-bead-nucleic-acid structures.

Four tubes of nucleic-acid solutions with concentrations of 2.5 μM, 5 μM, 7.5 μM and 10 μM were prepared to measure their absorbance. Four tubes of 100 μL of MBs solution were removed and washed twice with PBS. The supernatant was discarded, and different concentrations of nucleic-acid solutions were added to each MB. After a period of mixing, the nucleic acid was linked to the MB by a reaction between streptavidin and biotin. The absorbance of the residual supernatant after magnetic separation was measured. Comparing the difference in the absorbance of the solution before and after coupling, the beads reached saturation in a nucleic-acid solution at a concentration of 7.5–10 μM (Figure 2G,H). To ensure the coupling efficiency of the beads, we chose a nucleic-acid concentration of 10 μM as the optimum concentration.

### 3.4. Affinity Characterization of ZEN Toxin and Aptamer Chain

**Figure 3:**
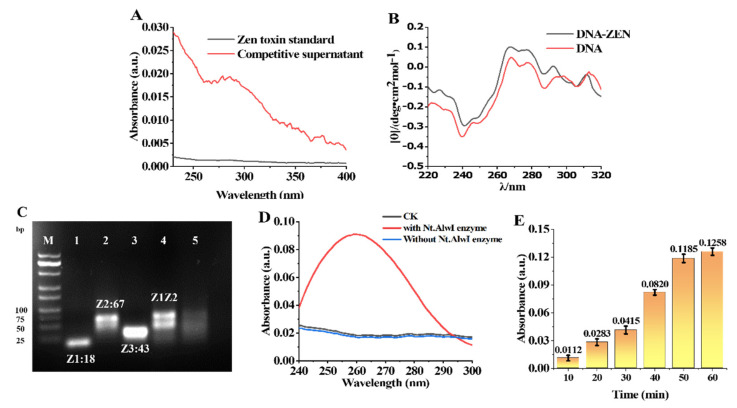
Verification of affinity of ZEN toxin for aptamer Z0 and characterization of Nt.AlwI Enzymes. (**A**) UV-Vis absorption spectra of the ZEN toxin standard solution (black curve) and the supernatant after Z0Z1 reacted with ZEN toxin (red curve). (**B**) Circular dichroism spectra of DNA (Z0Z1) and DNA-ZEN, and the affinity of ZEN for aptamer Z0. (**C**) Electrophoresis of 3% agarose gel to characterize the feasibility of Nt.AlwI digestion, lane M: DNA marker, lane 1: Z1, lane 2: Z2, lane 3: Z3, lane 4: Z1Z2, and lane 5: Nt.AlwI-digestion product. (**D**) CK: Ultraviolet absorption spectrum of the solution before reaction. Ultraviolet absorption spectrum of the upper liquid after a full reaction (blue curve without Nt.AlwI enzyme and red curve with Nt.AlwI enzyme). (**E**) UV-Vis absorption spectra of the Nt.AlwI enzyme digestion in 10–60 min.

The affinity of the ZEN toxin for the aptamers was verified by absorbance via UV-visible spectroscopy (Figure 3A). The prepared MB-Z0Z1 was added to a sample solution (100 μL) to be tested, rotationally mixed, and fully reacted at room temperature. The ZEN toxin bound to Z0 and the Z1 sequence was released. The supernatant containing Z1 was obtained by the magnetic separation of the solution after the reaction. The Z1 supernatant was measured by UV-Vis spectroscopy. It can be seen from the change in the value at 260 nm before and after the reaction that the aptamer had an affinity for the ZEN toxin. The change in DNA conformation was closely related to the change in the circular dichroism (CD) signal of the DNA. The DNA has a positive peak near 260–280 nm, which was caused by the stacking of bases, while the peak of DNA-ZEN was enhanced near 260–280 nm (Figure 3B). This is because the DNA was composed of two ssDNAs of different lengths, in which the Z0 aptamer chain contained 62 bases and its partially complementary Z1 ssDNA contained 18 bases, of which only 11 were complementary to Z0. ZEN had a high affinity for the Z0 aptamer; when it interacted with the Z0Z1 DNA, Z0 separated from Z1 and then folded on itself to trap ZEN, resulting in more base stacking.

### 3.5. Characterization of Nt.AlwI Enzyme

First, 10 μL of the Z1 ssDNA and Z2 ssDNA solutions (20 μM) were added, followed by 4 μL of 10× CutSmart Buffer and 16 μL of enzyme-free water. After the hybridization reaction, Z1Z2 dsDNA (5 μM) was obtained. Nucleic-acid solutions of Z1 and Z2, as well as Z3 ssDNA (the product of dsDNA Z1Z2 digested by Nt.AlwI) with the same concentrations were prepared under the same conditions. Half of the resulting Z1Z2 dsDNA (5 μM) solution was removed, and 1 μL of Nt.AlwI (1 μM) was added and allowed to fully react at 37 °C for 1 h. The remaining half of Z1Z2 dsDNA (5 μM) and the other nucleic-acid solutions were reacted, respectively, under the same conditions for the same amount of time.

To verify whether the Nt.AlwI enzyme has enzymatic activity on the designed DNA, the results were characterized by 3% agarose electrophoresis. Lane M: DNA marker, lane 1: Z1, lane 2: Z2, lane 3: Z3, lane 4: Z1Z2, and lane 5: Nt.AlwI-digestion product. As can be seen from lane 5, the Z2 strand of the Z1Z2 dsDNA was cleaved by the Nt.AlwI enzyme (Figure 3C).

To further verify the feasibility of the Nt.AlwI enzyme digestion of two self-assembled ssDNAs, a solution system containing MB-Z2 and a small amount of Z1 ssDNA was designed for experimental verification. A solution of 100 μL of the prepared MB-Z2 was aliquoted and the supernatant was removed through a magnetic rack. To the isolated MB-Z2 was added 1 μL of Z1 ssDNA (1 μM) and 99 μL of CutSmart buffer. The solution was mixed and reacted at 37 °C for 10 min, then 1 μL of Nt.AlwI enzyme (1 μM) was added and reacted for 60 min. The results are shown in the UV-Vis absorption spectrum (Figure 3D). At 260 nm, the value of the solution raised significantly. It was proved that many nucleic acids were cleaved from MB-Z2 into the solution, which further verified the feasibility of the Nt.AlwI enzyme.

To further optimize the reaction time of Nt.AlwI, we used a UV spectrophotometer to measure the UV-absorption spectra of the solution from 10–60 min. Six aliquots of 100 μL of the prepared MB-Z2 solution were taken out and the supernatant was removed through a magnetic rack. To the isolated MB-Z2 was added 1 μL of the Z1 ssDNA (1 μM) and 99 μL of CutSmart buffer. The solution was mixed and reacted at 37 °C for 10 min, then 1 μL of Nt.AlwI enzyme (1 μM) was added and reacted for 10–60 min. Therefore, 30 min was selected as the reaction time to prevent the reaction time from being too long and the detection limit from being too low (Figure 3E).

### 3.6. Evaluation of the Optimized Conditions for Trans-Cleavage

**Figure 4:**
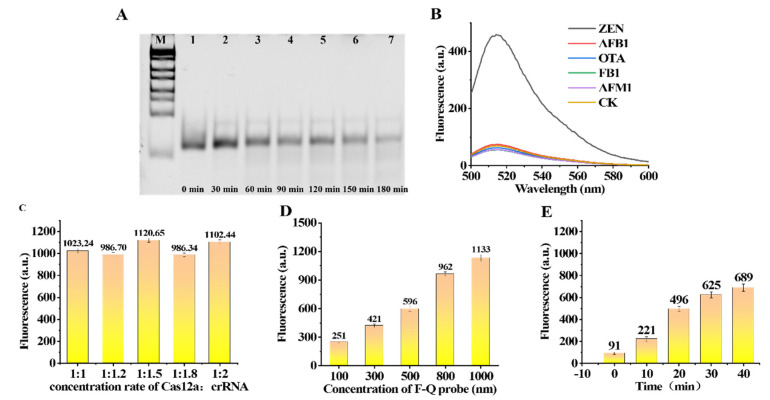
Characterization of Cas12a. (**A**) Seven small centrifuge tubes were prepared, and to each tube was added 10 μL of 5 μM ssDNA R1 (40 nt) as a substrate for trans-cleavage by the Cas enzyme, and to tubes 2–7 was added the same amount of Cas12a-crRNA-Z3 enzyme, and the reaction was carried out at 37° for 30–180 min, respectively. Lane M was a DNA marker, and the reaction product was subjected to 10% polyacrylamide electrophoresis. (**B**) Under the same conditions, the integrity of Cas enzyme on the effect of digestion. a: Quenched Reporter + Cas12a-crRNA + Z3; b: Quenched Reporter + Cas12a; c: Quenched Reporter + Cas12a-crRNA; d: Quenched Reporter. (**C**) The fluorescence intensity of the trans-cleavage activity of Cas12a-crRNA at four concentration ratios. (**D**) The fluorescence intensity of Quenched Reporter (concentrations of 100 nM, 300 nM,500 nM, 800 nM, and 1000 nM) when the fluorescence signal was restored after it was completely cut by CRISPR-Cas12a. (**E**) The fluorescence intensity of the Cas12a after cutting for 0–40 min.

Solutions of 7 μL of Cas12a (1 μM) and 7 μL of crRNA (1 μM) were added to enzyme-free PCR tubes, followed by 7 μL 10× NEBuffer™2.1 and 42 μL of RNase-free water. Then, 7 μL of Z3 ssDNA (1 μM) was added. The solution continued to react for ten minutes to obtain a solution of the Cas12a-crRNA-Z3 enzyme complex. A solution of 10 μL of ssDNAR1 (40 nt) was added to each of the seven enzyme-free tubes. Then, 10 μL of the solution of Cas12a-crRNA-Z3 enzyme complex and 1 μL of 10× NEBuffer™2.1 were added to each tube. Finally, seven solutions were reacted for 0 min, 30 min, 60 min, 90 min, 120 min, 150 min and 180 min, respectively. To verify whether the Cas12a-crRNA-Z3 enzyme complex can be successfully assembled and have enzymatic activity, we carried out verification through 10% polyacrylamide gel electrophoresis. The Cas12a-crRNA-Z3 enzyme complex can efficiently cleave ssDNA (Figure 4A).

To verify that the designed crRNA and Z3 can match the Cas12a enzyme used in the experiment, we designed a small experiment to verify. Solution a: 1 μL of the Cas12a enzyme (1μM) and 1 μL of crRNA (1 μM) were incubated for 10 min at room temperature. Then, 1 μL of Z3 ssDNA (1 μM) was added to the solution and incubation continued for 10 min. Finally, 20 uL of the Quenched Reporter was added to the solution. The buffer was NEBuffer™ 2.1. The solution system was reacted for 1 h at 37 °C and the fluorescence intensity of the solution was measured. Solution b: 1 μL of the Cas12a enzyme (1 μM) and 20 uL of the Quenched Reporter were added to the solution. The buffer was NEBuffer™ 2.1. The solution system was reacted for 1 h at 37 °C and the fluorescence intensity of the solution was measured. Solution c: 1 μL of the Cas12a enzyme (1 μM) and 1 μL of crRNA (1 μM) were incubated for 10 min at room temperature. Finally, 20 uL of the Quenched Reporter was added to the solution. The buffer was NEBuffer™ 2.1. The solution system was reacted for 1 h at 37 °C and measured the fluorescence intensity of the solution. Solution d: 20 uL of the Quenched Reporter was added to the solution. The buffer was 200 μL of NEBuffer™ 2.1. The solution system was reacted for 1 h at 37°C and the fluorescence intensity of the solution was measured. The final concentration of the substance was the same in all the solutions. As a result, the designed nucleic-acid sequence was correct and effective, and it was only after the formation of the complete Cas12a-crRNA-Z3 complex that the Cas12a-crRNA-Z3 enzyme cleaved the Quenched Reporter ssDNA and released fluorescence (Figure 4B).

The Cas12a-crRNA duplex was formed in NEBuffer™ 2.1. The Cas12a-crRNA complex was formed after standing for about 10 min at room temperature. To further study the ratio of Cas12a and crRNA, we carried out five experiments with ratios from 1:1 to 1:2. At different concentration ratios, Cas12a efficiently binds to crRNA by forming Cas12a-crRNA duplexes and the trans-cleavage activity was not significantly different (Figure 4C). Therefore, to reduce the cost, we chose a Cas12a:RNA ratio of 1:1 for the following experiments. To roughly evaluate the shearing efficiency of the CRISPR-Cas12a, we further evaluated the trans-cleavage performance of the CRISPR/Cas enzyme to cleave the Quenched Reporter at different concentrations. Five concentrations of the Quenched Reporter of 100 nM, 300 nM,500 nM, 800 nM and 1000 nM were designed to be the substrate, and the fluorescence intensity generated by trans-cleavage was proportional to the concentration of the substrate (Figure 4D). Finally, considering the difference and cost savings of the experiment, the Quenched Reporter with a concentration of 500 nM was selected for the experiment.

To obtain a suitable reaction time, we used a 5 nM concentration of the Cas12a-crRNA-Z3 complex to cleave a 500 nM concentration of the Quenched Reporter in a total volume of 200 μL. The fluorescence intensity was measured from the time when the Quenched Reporter was added. The fluorescence intensity readings were obtained at 0, 10, 20, 30, and 40 min (Figure 4E). Finally, we selected 20 min as the reaction time for the experiment.

### 3.7. Sensitivity Detection of the Fluorescence Aptasensor

**Figure 5:**
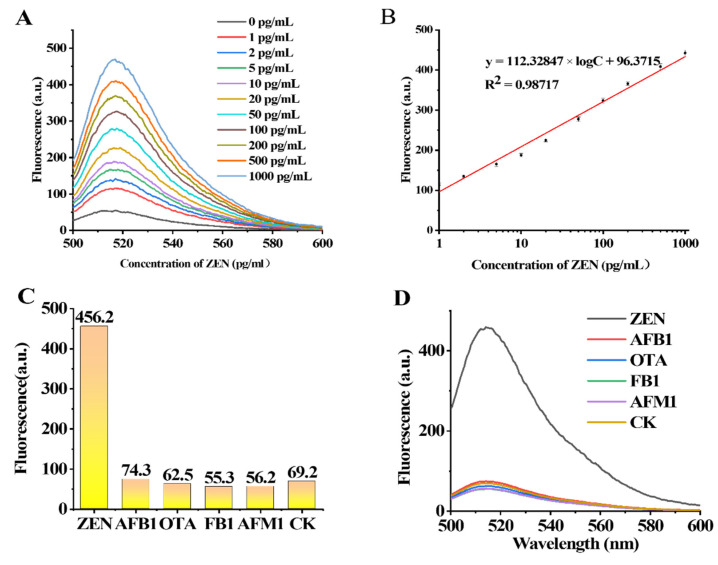
Characterization of fluorescent aptamer sensors for specific and sensitive detection. (**A**,**B**) Fluorescence emission spectra with different concentrations of ZEN (0 fg/mL, 1 fg/mL, 10 fg/mL, 100 fg/mL, 1 pg/mL, 10 pg/mL, 100 pg/mL and 1 ng/mL). (**C**,**D**) Specificity evaluation of this fluorescence aptasensor for ZEN.

To verify the sensitivity of the designed aptasensor to different concentrations of ZEN, experiments were carried out with different concentrations of ZEN (0 pg/mL, 1 pg/mL, 2 pg/mL, 5 pg/mL, 10 pg/mL, 20 pg/mL, 50 pg/mL, 100 pg/mL, 200 pg/mL, 500 pg/mL and 1000 pg/mL). The change in ZEN concentration in the range of 1 pg/mL–1000 pg/mL was positively correlated with the fluorescence intensity measured at 517 nm (Figure 5A,B). The regression equation of ZEN was y = 112.32847 × X + 96.3715 (X = log [CZEN] (pg/mL)), R^2^ = 0.98717. The LOD was calculated to be 0.213 pg/mL according to the 3*SD/slope (SD is the standard deviation of the blank sample, and slope is the slope of the standard curves). We summarized and compared the sensors for ZEN detection reported in recent years and found that the sensor in this experiment has higher sensitivity (Table S3).

### 3.8. Specificity Detection of the Fluorescence Aptasensor

Four kinds of mycotoxins including OTA, FB1, AFB1 and AFM1 were selected as the interfering mycotoxins with which to verify the specificity of the method for the simultaneous detection of ZEN toxins. The concentration of each toxin was 100 pg/mL. The specificity results are shown in Figure 5C,D. The fluorescence value shows that the ZEN toxin value is significantly higher than that of the other toxins; Therefore, the detection method has a high specificity for the ZEN toxin.

### 3.9. Monitoring of ZEN in Real Sample

In this experiment, corn oil was chosen to verify the detection performance of the ZEN toxin in actual samples. The samples containing three different concentrations of ZEN (1, 50, 100 pg/mL) were prepared from the blank corn oil. The identification probe MB-Z0Z1 was prepared as in step 2.4. A re-suspension solution of 10 μL of MB-Z0Z1 was aliquoted, and the supernatant was removed after the magnetic separation of the solution. Then, the MB-Z0Z1 precipitate was retained. A sample solution of 100 μL containing ZEN was added to MB-Z0Z1 and shaken at room temperature for 30 min. The solution after the reaction was subjected to magnetic separation, and the supernatant was reserved at 4 °C for later use. Amplification probe MB-Z2 was prepared as described in step 2.6. A solution of 100 μL of MB-Z2 was magnetically separated, the supernatant was removed, and the precipitated MB-Z2 was retained. The preserved supernatant was added to the MB-Z2 precipitate and then reacted at 37 °C for 10 min. A solution of 1 μL of Nt.AlwI enzyme (1 μM) and 20 μL of 10× CutSmart buffer were added to the solution, and the solution was diluted to 200 μL with enzyme-free water, then the reaction continued for 30 min. At the end of the reaction, the liquid in the solution was separated by a magnetic rack, and the enzyme was inactivated at 80 °C for 20 min and then quickly cooled to 4 °C for later use. The Cas12A-crRNA complex was prepared according to Step 2.8 and added to the preserved solution, followed by 30 uL 10X NEBuffer 2.1. The solution reacted for 10 min at room temperature, then 15 μL of the Quenched Reporter (10 μM) was added to the solution and the solution was diluted to 300 μL with enzyme-free water. The solution reacted for 20 min at 37 °C. After that reaction, the solution was inactivated at 65 °C for 10 min. Finally, the fluorescence intensity of the solution was measured. The detection-effect data of the verification sensor was obtained (Table 1). The detection rate of ZEN in the corn-oil samples was 94.18–106%.

## 4. Conclusions

Here, we designed a dual-enzyme-assisted signal-amplification aptasensor for the specific monitoring of ZEN. The magnetic beads act as a bridge in the amplification of the two signals connecting the Nt.AlwI enzyme and the Cas12a enzyme. The concentration of ZEN added was determined by the fluorescence intensity at 517 nm. This method can monitor ZEN in the linear range of 1 pg/mL–1 ng/mL, and the detection limit was as low as 0.213 pg/mL. In addition, the aptasensor showed a good recovery rate in the corn-oil samples.

The research ideas of this experiment can be applied to the detection of target substances in various complex components, such as pesticide residues, various toxic and harmful components, etc. It can also be used in the detection of liquid mixtures with many complex components.

In the testing process of this sensor, excessive detection probes can be limited with ZEN and can achieve toxin separation through magnetic separation, which greatly improves the safety of the testing process. Next, we improved the sensor to add an enrichment function in the Z1 and Z2 combination step to achieve the application of the sensor to large-scale sample detection.

For small molecular targets, the aptamer signal-amplification strategy is a reliable way to eliminate the detection limitations and improve the detection sensitivity of biosensors. Although there are still some aspects to be improved, the excellent extensibility of aptamers makes its development potential endless.

## Figures and Tables

**Table 1:** The real detection of ZEN at different concentrations in food samples.

Sample	Added (pg/mL)	Found (pg/mL)	Recovery (%)
1	1	1.06 ± 2.34	106%
2	50	47.09 ± 11.69	94.18%
3	100	101.27 ± 14.78	101.27%

## Data Availability

Data is contained within the article (or supplementary material).

## Supplementary Materials

The following are available online at https://www.mdpi.com/article/10.3390/foods11030487/s1, Table S1: Sequences involved in this study, Table S2: Composition of different reaction buffer, Table S3: Reported LOD for ZEN detection. References [6,29,30,31,32] are cited in the supplementary materials.
