# Supplementary material for: Dual-Enzyme-Based Signal-Amplified Aptasensor for Zearalenone Detection by Using CRISPR-Cas12a and Nt.AlwI
Source: Foods. 2022 Feb 8;11(3):487. doi: 10.3390/foods11030487 (PMC8834192; doi:10.3390/foods11030487)
Supplement: Supplementary file 1 [file foods-11-00487-s001.zip › foods-1528993-supplementary.pdf]

Supplementary Materials

Table S1. Sequences involved in this study

**Name**	**Sequence (5'-3')**
Z0	/5`Biotin/CTACCAGCTTTGAGGCTCGATCCAGCTTATTCAATTA TACCAGCTTATTCAATTATACCAGC
Z1	GCTGGTATAATTAGATCC
Z2	/5`Biotin/ATACTCGATATCCAGGGATCTAAT▼ TATACCAGCGCGCAGCGTGCTCAGCGAAGGGAAAGGATGTAG G
Z3	TATACCAGCGCGCAGCGTGCTCAGCGAAGGGAAAGGATGTAG G
crRNA	UAAUUUCUACUAAGUGUAGAU**CCUUCGCUGAGCACGCUGC GC**
Quenched Reporter	/6-FAM/AAAAAAAAAA/BHQ1/
R1	AAAAAAAAAAAAAAAAAAAAAAAAAAAAAAAAAAAAAAAA

Table S2. Composition of different reaction buffer

**Buffer name**	**Composition**
NEB CutSmart™ buffer	50 mM KAc, 20 mM Tris-Ac, 10 mM Mg(Ac)2, 100 µg/ml BSA（pH 7.9 @ 25℃）
NEBuffer™ 2.1	50 mM NaCl, 10 mM Tris-HCl, 10 mM MgCl2, 100 µg/ml BSA（pH 7.9 @ 25℃）
Buffer 1	10 mM Tris-HCl, 1 mM EDTA, 100mM NaCl, 0.01% -0.1% Tween-20

Table S3. Reported LOD for ZEN detection

Recognition element	Transduction assay	LOD	Linear range	Reaction time	Ref.
anti-idiotypic nanobodies	Phage display mediated immuno-polymerase chain reaction (PD-IPCR)	0.09 ng/ mL	0.43-14.2 ng/mL	Within 2 days	[6]
Molecularly imprinted polymers	Cyclic voltammetry and electrochemical impedance spectroscopy	0.2 ng/mL	2.5-200 ng/ mL	Within 2 hours	[29]
Colloidal Au sphere (SP) and nanorods (NRs)	Lateral flow immunochromatographic assays (ICAs)	3.0 μg/L and 5.0 μg/L	none	Within 10 min	[30]
Aptasensor	Square wave voltammetry	0.017 ng/mL	0.01-1000 ng/mL	Within 2 days	[31]
Aptasensor	Fluorescence based on CQDs	7.5 nM	31.4-628 nmol/L	Within 2 hours	[32]
Aptasensor	Fluorescence	0.213 pg/mL	1-1000 pg/mL	Within 2 hours	This study
